# Supplementary material for: Automated Road Defect and Anomaly Detection for Traffic Safety: A Systematic Review
Source: Sensors (Basel). 2023 Jun 16;23(12):5656. doi: 10.3390/s23125656 (PMC10305190; doi:10.3390/s23125656)
Supplement: Supplementary file 1 [file sensors-23-05656-s001.zip › sensors-2327788-supplementary.pdf]

A Modified PRISMA Checklist for Systematic Reviews in ARDAD Research

The Preferred Reporting Items for Systematic Reviews and Meta-Analyses (PRISMA) checklist [1], as shown below, is adapted to the field of Automated Road Defects and Anomaly Detection (ARDAD) research as part of the research article titled "Automated Road Defect and Anomaly Detection for Traffic Safety: A Systematic Review".

The modifications consider the extension-specific characteristics of ARDAD research, such as the use of computer vision, the diversity of data sources, and the rapid evolution of the field to improve the quality and transparency of systematic reviews and facilitate the synthesis of evidence for future research and practice.

We encourage further feedback and suggestions.

**Section and Topic**	**Item No.**	**Description**
Title	1	- Identify the report as a systematic review in the title.<br>- Use an informative title that clearly states the review's main objectives or research questions related to ARDAD techniques or applications.<br>- Consider including additional information in the title, such as the specific ARDAD method or application under investigation, the type of data being analysed, or any unique aspects of the review process (e.g., update of a previous review, use of a living systematic review approach).
Abstract	2	- The abstract should clearly state the systematic review's main objective(s) or research question(s). It should also provide essential information about the review's methods, findings, and implications to help readers decide whether to access the full report.<br>- Results must be reported for all primary outcomes related to the main review objective(s) or question(s), regardless of statistical significance, magnitude, or direction of effect.<br>- Keywords used in the abstract should accurately describe the review question and its findings and be used to index the

		review in bibliographic databases. It is recommended to include terms that describe the real-world problem addressed, the proposed ARDAD solution, and the results obtained.
Introduction	3<br><br><br><br><br><br><br><br><br><br><br><br><br><br><br><br><br><br>4	Rationale<br>- Describe the current state of knowledge and uncertainties in the field of ARDAD and how it relates to the problem being addressed.<br>- Articulate why the intended review is important in the context of ARDAD research.<br>- If other systematic reviews addressing the same or similar questions are available, explain why the current review is necessary and how it differs from existing reviews. If the review is an update or replication of a previous review, indicate this and cite the previous review.<br>- If the review examines the effects of ARDAD techniques or algorithms, briefly describe how these techniques or algorithms are expected to work.<br>- If the ARDAD solution or context is complex in its delivery, present a logical model to visually display the hypothesied relationship between solution components and outcomes.<br><br>Objectives<br>- Provide an explicit statement of all objectives or questions the review addresses, expressed in terms of a relevant question formulation framework specific to ARDAD research.<br>- If the purpose is to evaluate the effects of an ARDAD solution, use the Problem, Solution, Comparison, Results (PSCR) framework or one of its variants to state the comparisons made.
Methods	5	Eligibility Criteria<br>- Specify all study characteristics used to decide whether a study was eligible for inclusion in the review, that is, components described in the PSCT framework or one of its variants, and other characteristics, such as eligible study design(s) and setting(s), and minimum duration of follow-up.<br>- Specify eligibility criteria concerning report characteristics, such as year of dissemination, language, and report status (e.g., whether reports, such as unpublished manuscripts and

	6<br>7	conference abstracts, were eligible for inclusion).<br>- Indicate if studies were ineligible because the outcomes of interest were not measured or ineligible because the results for the outcome of interest were not reported.<br>- Specify any groups used in the synthesis (e.g., ARDAD technique, outcome and dataset groups) and link these to the comparisons specified in the objectives (item No. 4).<br>- Consider providing rationales for any notable restrictions to study eligibility, such as excluding studies with a low-quality dataset or studies using outdated techniques.<br>Information Sources<br>- Specify the date when each source (e.g., database, library, website, organisation) was last searched or consulted.<br>- If bibliographic databases were searched, specify for each database its name (e.g., Springer or IEEE), the interface or platform through which the database was searched (e.g., Scopus, Web of Science), and the dates of coverage (where this information is provided).<br>- If study registers, regulatory databases and other online repositories were searched, specify the name of each source and any date restrictions applied.<br>- If websites, search engines or other online sources were browsed or searched, specify the name and URL of each source.<br>- If organisations or software developers were contacted to identify studies, specify the name of each source.<br>- If individuals were contacted to identify studies, specify the types of individuals contacted (e.g., authors of studies included in the review or researchers with expertise in the area).<br>- If reference lists were examined, specify the types of references examined (e.g., references cited in study reports included in the systematic review or references cited in systematic review reports on the same or similar topic).<br>- If cited or citing reference searches (also called backward and forward citation searching) were conducted, specify the bibliographic details of the reports to which citation searching was applied, the citation index or platform used (e.g., Web of Science), and the date the citation searching was performed.<br>- If journals or conference proceedings were consulted, specify the names of each source, the dates covered, and how they

	8	were searched (e.g., hand-searching or browsing online).<br>Search Strategy<br>- Provide the complete search strategy used in each database or interface, including the search terms and any Boolean operators or truncation used.<br>- Explain any limitations placed on the search, such as language or date range, and provide a justification for these limitations based on the eligibility criteria of the review.<br>- If previously published search strategies or search filters were used, provide a citation, and describe any adaptations made to them.<br>- If natural language processing or text analysis tools were used to refine the search terms, specify the tools used.<br>- If a translation tool was used to translate search strings from one language to another, specify the tool used.<br>- If the search strategy was validated, describe the validation process used and specify which studies were included in the validation set.<br>- If the search strategy was peer-reviewed, report the peer review process used and any tools used, such as the Peer Review of Electronic Search Strategies (PRESS) checklist.<br>- If a PSCT-style approach was not used, describe the final conceptual structure and any explorations undertaken to achieve it.<br><br>Selection Process<br>Recommendations for reporting regardless of the selection processes used:<br>- Report how many reviewers screened each record (title/abstract) and each report retrieved, whether multiple reviewers worked independently at each screening stage or not, and any processes used to resolve disagreements between screeners.<br>- Report any processes used to obtain or confirm relevant information from study investigators.<br>- If abstracts or articles required translation into another language to determine their eligibility, report how these were translated.<br><br>Recommendations for reporting in systematic reviews using automation tools in the selection process:<br>- If crowdsourcing was used to screen records, provide details

	9	of the platform used and specify how it was integrated within the overall study selection process.<br>- If datasets of already-screened records were used to eliminate records retrieved by the search from further consideration, briefly describe the derivation of these datasets.<br><br>Data Collection Process<br>- Report how many reviewers collected data from each report, whether multiple reviewers worked independently or not, and any processes used to resolve disagreements between data collectors.<br>- Report any processes used to obtain or confirm relevant data from study investigators.<br>- If any automation tools were used to collect data, report how the tool was used, how the tool was trained, and what internal or external validation was conducted to understand the risk of incorrect extractions.
	10	Data Items (outcomes)<br>- List and define all outcomes for which data were sought, including primary and secondary outcomes, and any subgroup analyses. Specify whether all results that were compatible with each outcome domain in each study were sought (e.g., for all measures, time points, analyses), and if not, the methods used to decide which results to collect.<br>- List and define all other variables for which data were sought (e.g., participant and intervention characteristics, study design, funding sources). Describe any assumptions made about any missing or unclear information, and any imputation methods used.<br>- Specify any data transformations or calculations performed on the collected data (e.g., calculation of effect sizes or standard errors).<br>- If meta-analyses or other types of synthesis were conducted, describe the methods used to combine and summarise the data, including any statistical models or software used.<br>- If sensitivity analyses were conducted to examine the robustness of the findings to different assumptions or decisions, describe these and report the results.
	11	Study Risk of Bias Assessment

		- Specify the methods used to assess the risk of bias in the included studies, including details of the tool(s) used, how many reviewers assessed each study and whether they worked independently. If applicable, provide details of any automation tools used in the process and how their performance was validated.<br>- Describe how the risk of bias assessment was conducted for studies of different designs (e.g., randomised controlled trials, observational studies, case studies) and how any relevant domain-specific guidance was applied.<br>- Indicate which aspects of study quality (e.g., risk of bias, precision, consistency) were taken into account when assessing the overall certainty of the evidence for each outcome, and how the assessment was conducted.<br>- Provide a summary of the risk of bias assessments for each included study or group of studies, and how this information was used in the synthesis (e.g., subgroup analyses, sensitivity analyses).
	12	Effect Measures<br>- Specify for each outcome the effect measure(s) (e.g., accuracy, precision, recall, F1-score) used in synthesising or presenting results.
	13	Synthesis Methods<br>- Describe the processes used to decide which studies were eligible for each synthesis process, such as tabulating the study intervention characteristics and comparing them against the planned groups for each synthesis process (item No. 5).<br>- Describe any methods required to prepare the data for presentation or synthesis, such as handling missing summary statistics or data conversions.<br>- Describe any methods used to tabulate or visually display results of individual studies and syntheses.<br>- Describe any methods used to synthesise results and provide a rationale for the choice(s). If meta-analysis was performed, describe the model(s), method(s) to identify the presence and extent of statistical heterogeneity, and software package(s) used.<br>- Describe any methods used to explore possible causes of heterogeneity among study results, such as subgroup analysis and meta-regression.

		- Describe any sensitivity analyses conducted to assess the robustness of the synthesised results."
	14	Reporting Bias Assessment<br>- Describe any methods used to assess the risk of bias due to missing results in the synthesis process (e.g., publication bias, selective outcome reporting bias).
	15	Certainty Assessment<br>- Describe the methods used to assess the certainty or quality of the evidence for each outcome, including the criteria used and the processes for rating or grading the certainty of evidence.<br>- Specify the tool(s) or framework(s) used to assess certainties, such as the GRADE approach or the Cochrane Risk of Bias tool for randomised trials.<br>- Describe any factors considered in downgrading or upgrading the certainty of the evidence, such as the risk of bias, inconsistency, indirectness, imprecision, or publication bias.<br>- Specify the rating or grading system used for the certainty of the evidence, such as high, moderate, low, or very low, and provide a justification for each rating or grade assigned.
	16	Study Selection<br>- Ideally, using a flow diagram, describe the search and selection process results, from the number of records identified in the search to the number of studies included in the review.<br>- Cite studies that might appear to meet the inclusion criteria but which were excluded, and explain why they were excluded.
	17	Study Characteristics<br>- Cite each included study and provide its characteristics, such as study design, setting, sample size, outcomes assessed, and length of follow-up.<br>- Specify whether each study is included in the synthesis and, if not, explain why.<br>- If meta-analysis was performed, provide forest plots (or equivalent) and, for each outcome, a summary of the results across studies, including estimates of effect size and precision (such as confidence intervals), and a rating of the certainty of

		the evidence (such as GRADE).<br>- If meta-analysis was not performed, provide a narrative summary of the results across studies for each outcome, taking into account the risk of bias, imprecision, inconsistency, and other relevant factors, such as the presence of publication bias or selective outcome reporting.
	18	Risk of Bias in Studies<br>- Present assessments of risk of bias for each included study.
	19	Results of Individual Studies<br>- For all outcomes present, for each study: (a) summary statistics for each group (where appropriate) and (b) an effect estimate and it's precision (e.g. confidence/credible interval), ideally using structured tables or plots.
	20	Results of Syntheses<br>- Briefly summarise each synthesis's characteristics and risk of bias among contributing studies.<br>- Present results of all statistical syntheses conducted. If meta-analysis was conducted, present for each the summary estimate and its precision (e.g. confidence/credible interval) and measures of statistical heterogeneity. If comparing groups, describe the direction of the effect.<br>- Present results of all investigations of possible causes of heterogeneity among study results.<br>- Present results of all sensitivity analyses conducted to assess the robustness of the synthesised results.
	21	Reporting Biases<br>- Describe any methods used to assess the risk of bias due to missing results in a synthesis (arising from reporting biases).
	22	Certainty of Evidence<br>- For each outcome assessed in the review, present an assessment of the certainty (or confidence) in the body of evidence. This assessment should be based on the quality of the studies included, the consistency of the findings, the precision of the effect estimates, the potential for publication bias, and any other relevant factors.<br>- If there are differences in the certainty of evidence across outcomes or subgroups, provide a clear explanation for these differences.<br>- For ARDAD research, special attention may need to be paid

		to the quality of the data used in the analysis, such as the accuracy and completeness of annotations, potential sources of bias in the data collection process, and the potential impact of algorithmic bias. These factors may be particularly relevant for studies that rely heavily on machine learning or other forms of artificial intelligence.
	23	Discussion<br>- Provide a general interpretation of the results of the review, in the context of other evidence in the field of ARDAD.<br>- Discuss any limitations of the evidence included in the review, such as the quality of the studies or the limitations of the techniques used in the studies.<br>- Discuss any limitations of the review processes used, such as limitations in the search strategy or selection process.<br>- Discuss the implications of the results for practice, policy, and future research in the field of ARDAD, including any recommendations for further research or applications in real-world scenarios.
	24	Registration and Protocol<br>- Provide registration information for the review, including the register name and registration number, or state that the review was not registered.<br>- Indicate where the review protocol can be accessed or state that a protocol was not prepared.<br>- Describe any amendments to the information provided at registration or in the protocol.
	25	Support<br>- Describe sources of financial or non-financial support for the review and the role of the funders or sponsors in the review.
	26	Competing Interests<br>- Declare any competing interests of review authors.
	27	Availability of Data, Code, and Other Materials<br>- Report the availability and location of the following materials: template data collection forms, data extracted from included studies, data used for all analyses, analytic code, and any other materials used in the review related to ARDAD, such as trained models, datasets, or computer code for running experiments. If any of these materials are not publicly available, provide contact information for

		requesting access.

1. PRISMA. *Preferred Reporting Items for Systematic Reviews and Meta-Analyses*. 2020 10/01/2023]; Available from: http://www.prisma-statement.org/PRISMAStatement/Checklist.aspx.
